# Comprehensive Dataset on Electrical Load Profiles for Energy Community in Ireland

**DOI:** 10.1038/s41597-024-03454-2

**Published:** 2024-06-12

**Authors:** Rohit Trivedi, Mohamed Bahloul, Aziz Saif, Sandipan Patra, Shafi Khadem

**Affiliations:** International Energy Research Centre, Tyndall National Institute, UCC, Dyke Parade, Cork, Ireland

**Keywords:** Energy management, Photovoltaics

## Abstract

This paper describes a comprehensive energy-related dataset, collected from residential electricity households within an energy community in Ireland, as part of StoreNet project. The data includes local weather parameters and per household power (W) and energy (Wh) components for various aspects such as active power consumption, PV generation, grid import and export, charging and discharging, and the state of charge of energy storage. Additionally, it provides weather data for the location at a 1-minute temporal resolution for the year 2020. The dataset has been validated by comparing measurements that should yield identical results to standard load profiles, with no significant inconsistencies discovered. Validation examples have also been given through the published articles where this dataset has been used to analyse peer-to-peer energy trading benefits for the energy community and decision-making support for aggregators. The dataset aids in understanding patterns in electrical load curves and the duty cycle of energy storage within an energy community. Furthermore, it can assist in comprehending the impact on distribution networks caused by distributed energy storage.

## Background & Summary

Energy communities are groups of citizens who collectively organise and take action to transition to clean energy, increase public acceptance of renewable energy projects and attract private investments. Energy communities can benefit citizens by increasing energy efficiency, lowering electricity bills, and creating local job opportunities. The EU has introduced the concept of energy communities in its legislation, with new rules allowing for active consumer participation through citizen energy communities, renewable energy communities, and flexibility services^1^. The EU Solar Energy Strategy also mandates the establishment of at least one renewable energy community (REC) per municipality with over 10,000 residents by 2025. This is especially relevant given the high-priority concerns surrounding energy prices, supply security, and energy system resilience^2^.

According to a report from the Joint Research Centre of the EU, it is estimated that energy communities will own approximately 17% of installed wind capacity and 21% of solar by 2030^3^. By 2050, it is expected that nearly half of all households in the EU will produce renewable energy, which will account for 45% of electricity production^4^. Although energy communities may operate independently in certain locations, such as islands or remote areas, they will mostly remain connected to the energy system. Integrating energy communities into the energy system should be cost-efficient and provide value to all customers while also delivering real savings for the energy system as a whole.

The exchange and accessibility of data are vital to facilitate peer-to-peer (P2P) energy trading both within and beyond the energy community. This data pertains to the consumption and generation behaviour of various participants in the P2P trading scheme. A recent online experiment analysed the P2P trading choices of German homeowners, simulating the effects of various decision-making strategies on P2P communities. The research found that allowing prosumers to trade energy resulted in marginally higher levels of community self-sufficiency than when they solely prioritised maximising their self-consumption^5^. P2P trading relies on smart metering systems to collect this data. The European Commission previously estimated that nearly 200 million smart meters for electricity markets would be deployed in the EU by 2020. However, the Commission’s prediction was unmet, and several Member States encountered issues when rolling out smart meters. Additionally, P2P energy trading challenges both the traditional structure of the electricity system, which relies on a vertical supply relationship between a supplier and a user, and the legal framework for data management and data protection applied in general and sector-specific regulation^6^. Hence, collecting energy related data at the community level for further analysis and decision-making is becoming a great challenge.

There are several open-access datasets available that pertain to residential PV and storage systems. One such available dataset contains various PV generation and load scenarios in one-minute intervals^7^, but it does not include any data about battery storage operation. Another dataset, collected between 2006 and 2007, was published in^8^ and includes voltage, current, active and reactive power information for different sets of domestic appliances. A 15-minute resolution dataset is also available in^9^, which contains measurements from 370 residential households in Portugal between 2011 and 2014. Additionally, UK Power Networks published smart-meter data, with a 30-minute resolution, from over 5000 households measured between 2011 and 2014 in the UK^10^. The CoSSMic dataset^11^ includes electricity consumption profiles of 11 households in Konstanz, Germany, sampled at one-minute intervals between October 2013 and December 2016. Furthermore^12^, offers a dataset with a sampling rate of 15 Hz, which provides appliance-level energy consumption data for 22 households. Another dataset, which measures domestic electricity demand at a frequency of 1 Hz across 15 homes over a period of up to 3.5 years between 2016 and 2020, is publicly available. Lastly^13^, provides a dataset containing 96 days of aggregated and individual appliance energy consumption data for one household with 18 appliances, collected at a sampling rate of 0.5 Hz.

Although many datasets are available, they could be improved, especially the required datasets regarding local energy market modelling, community-based energy trading and microgrid solutions. The publicly available datasets lack important features with the same resolution that would help to understand the patterns of local weather, energy generation, consumption, battery storage operation, and interactions with external power grids in a single collected dataset. The StoreNet dataset can fill these gaps with its comprehensive combination of features, including large sample size, high temporal resolution, and numerous measured indicators.

## Methods

**Fig. 1:**
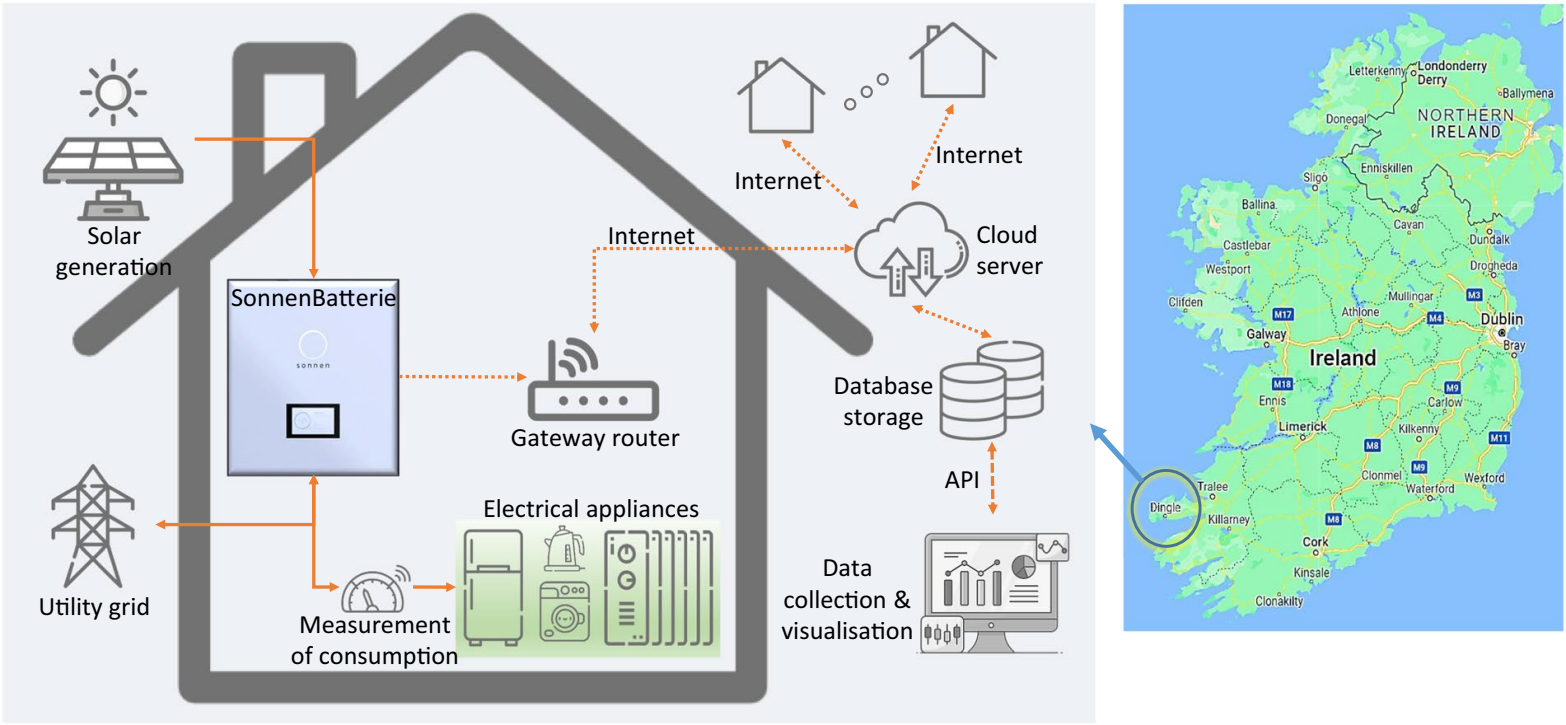
StoreNet project set-up.

**Fig. 2:**
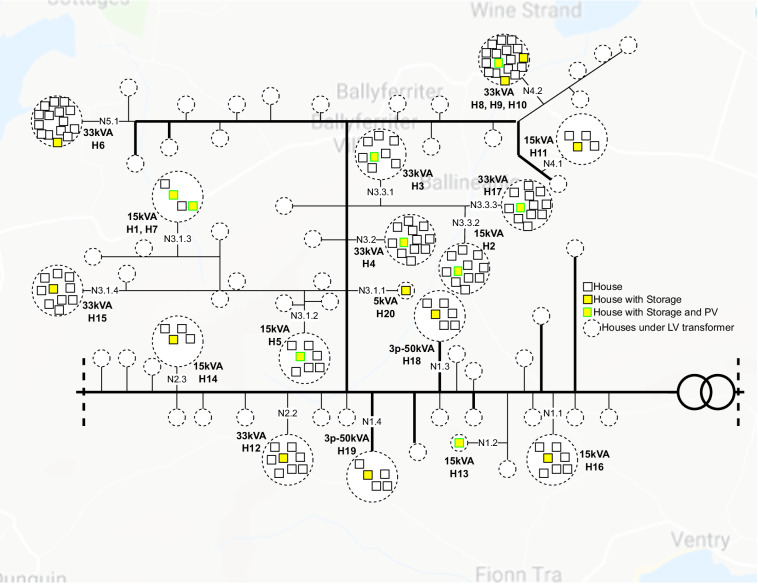
Simplified Network Diagram of Dingle Community showing the participating households (house with storage and house with storage and PV) in StoreNet project.

The “StoreNet” project is a collaborative effort led by the International Energy Research Centre (IERC). The consortium consists of IERC, Electric Ireland (a utility supplier), Solo Energy (an aggregator), and ESB Networks (a network operator). Its main goal is to demonstrate the effectiveness of residential energy storage solutions using batteries on the Irish grid while contributing to the knowledge of efficient transitioning to a sustainable energy future. This project aims to provide value to different stakeholders, including consumers, grid operators, and renewable generators. The trial took place on the Dingle Peninsula as part of ESB Network’s Dingle Electrification Project, which supports the Dingle 2030 initiative to enable an engaged energy community. Twenty houses were selected to participate in the demonstration, each equipped with a 3.3 kW/10 kWh residential Sonnen battery and a smart meter that considers day/night-time tariffs. However, only ten of these houses have installed rooftop solar PV systems with capacities ranging between 2–2.2 kWp. PV panels are mainly oriented around 35 degrees and mostly south-facing. Figure 1 displays the overall household system, including cloud communication, while Fig. 2 shows the location of the houses in the selected network (thick lines indicate 10 kV MV networks, whereas thin light lines are 3-phase 400 V), LV transformer rating and the number of houses under each transformer. The number of customers is almost evenly distributed within the 3-phase network. The authors have worked as part of the StoreNet project and directly contributed to conducting the trial, data collection, processing and analysing the outcomes.

**Fig. 3:**
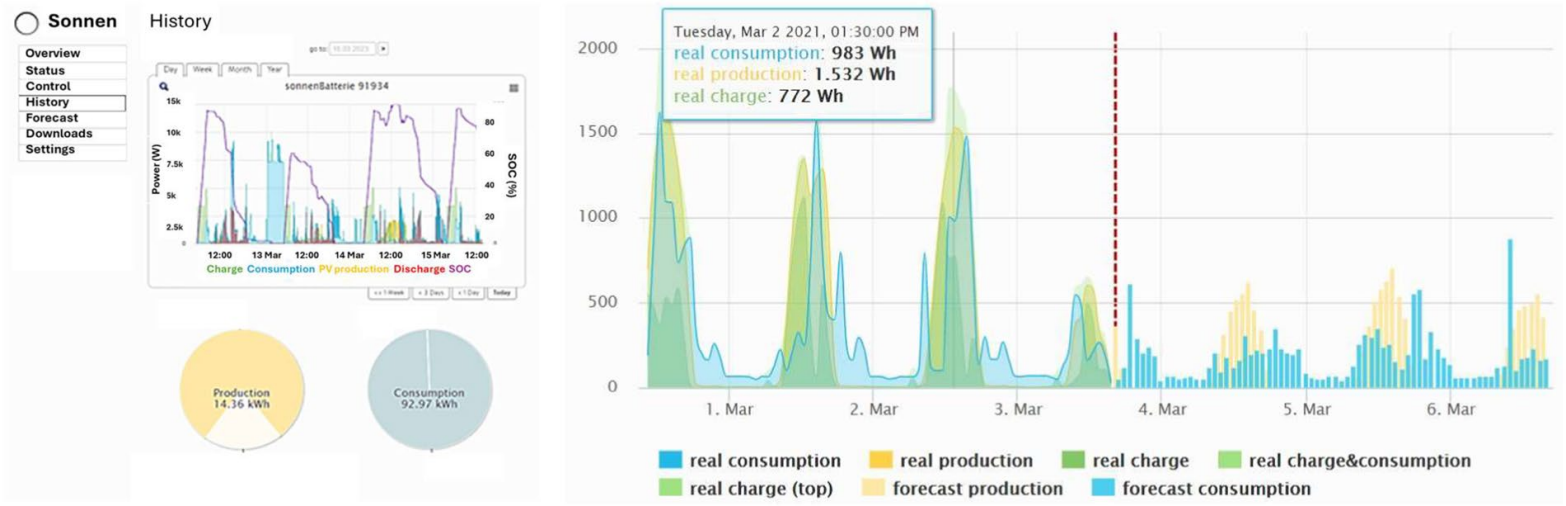
Sonnen dashboard for status display and data collection (**a**) real-time display; and (**b**) demand and generation forecast for three days.

The consortium has been able to gather various data about the operation of systems in the host network, thanks to SCADA systems. StoreNet uses a centralised control architecture, allowing the controller to determine the power exchange between the ESS, PV generators, houses, and the grid. Initially, Solo Energy (aggregator) created a basic self-consumption (SB-SC) algorithm and applied it to the initial operation design of the StoreNet platform. Although the functionality of this algorithm is described in the Sonnen manual^14^, it was not disclosed to the consortium due to IP and trade secret concerns. The Sonnen metering serves as the first layer of data acquisition in this project. The data collected from dashboard (Fig. 3a) in the StoreNet project is transmitted and stored in a cloud server through a gateway router. With the application programming interface (API), data was downloaded from the server and analysed for further use. Each house is tagged with an installation number to ensure anonymous data acquisition and an efficient GDPR policy implementation (91934 as an example) (Fig. 3b).

## Data Records

The data is readily available on figshare as a public repository^15^. Users can download the repository and conduct experiments on their local system. The data includes 41 CSV files in the ‘original data’ folder. Raw data in the ‘original data’ folder was processed using python script and the resulting processed data is stored in folder named ‘processed_data’ to perform data validation experiments. Among these files, 20 are for power measurements, and the other 20 are for energy measurements for each of the 20 houses with unique House IDs, ranging from H1 to H20. Additionally, there is one file that contains weather data for the entire year of 2020. For example, the files H1_W and H1_Wh contain the power and energy profiles of House 1, respectively. Additionally, a separate folder for plotting figures is generated, and Python code files are included to produce the figures presented in the paper. The file structure and data description for each file are detailed in Tables 1 and 2:

**Table 1:** Collected data structure.

Subfolder	Features	Resolution	Year	Comment
**Power**	Date, PV, Load, charging, Discharging, state of charge	1 minute	2020	Power profiles
**Energy**	Date, PV, Load, Import, Export, charging, Discharging, state of charge	1 minute	2020	Energy profiles
**Weather**	Date, Wind Speed, Direction, Temperature, Pressure, Total Radiation, Rainfall	1 minute	2020	Weather profiles

**Table 2:** Description of dataset columns.

	Feature	Description
**Power**	Date	Timestamps in 1-minute intervals (Year 2020)
	Production_W	Power generation from PV
	Consumption_W	Household consumption
	Charge_W	Battery storage charging
	Discharge_W	Battery storage discharging
**Energy**	Date	Timestamps in 1-minute intervals (Year 2020)
	Production_Wh	Energy generation from PV
	Consumption_Wh	Energy consumption
	Charge_Wh	Battery energy storage charging
	Discharge_Wh	Battery energy storage discharging.
	State-of-charge	Battery state of charge (%)
	From grid_Wh	Energy is taken from the power grid
	Grid feed_Wh	Energy delivered to the power grid
**Weather**	Date	Timestamps in 1-minute intervals (Year 2020)
	Speed	Wind speed (knots)
	Dir	Wind direction (degrees)
	Drybulb	drybulb temperature (degrees Celsius)
	Cbl	CBL pressure (hPa)
	Soltot	Solar radiation (J/cm2)
	Rain	rainfall (mm)

## Technical Validation

**Fig. 4:**
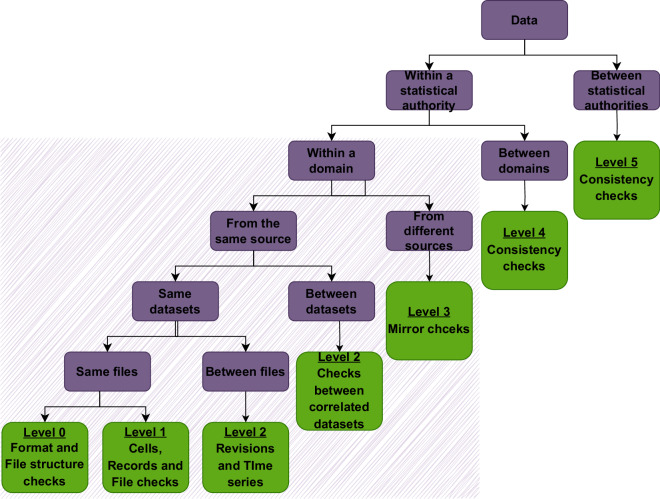
Data validation levels^16^.

This section utilises the EU-CROS (Collaboration in Research and Methodology for Official Statistics)^16^ validation-level approach to validate the structure and content of the dataset. The collected dataset methodology enables the validation of data up to levels 2 & 3 in accordance with the EU-CROS approach as shown in Fig. 4. The key points for these levels are explained below:

### Validation level 0

This level ensures that data is consistent with expected IT requirements. This level checks:

- Whether the file was sent or prepared by the authorised authority (data sender).
- Whether the column separator and end-of-record symbol are used correctly.
- Whether the file has the expected number of columns in the agreed format.
- Whether the columns have the expected format of the data, such as alphanumeric, numeric, etc.

For these quality checks, only the structure of the file or the format of the variables is necessary as input. Below is the basic sanity check done in this work:

**Table:** 

**Expected**	**Received**	**Remark**
File sent/prepared by authorised authority	✓	
8 columns (date, production, consumption, charge, discharge, feed-in, from-grid, state-of-charge) with proper respective units	✓	Units (Wh)
Proper format of each variable/column	✓	File information on first few rows to be removed
Length of the data (time period)	✓	In 1-min resolution

### Validation level 1

This level checks the consistency of data within the elements of the dataset. For these quality checks, only the statistical information included in the file itself is required. For example:

- Whether the number in columns is negative or not (as expected).
- Whether the year in the respective column matches the year in the file name.
- Whether there is consistency at the micro-level of two or more variables. For example, supply equals demand all the time.

Below is the basic sanity check done in this work:

**Table:** 

**Expected**	**Received**	**Remark**
Year in the file name corresponds to the same year in the data fields.	✓	
Records in the fields are positive integers with no categorical variable.	✓	All are integers
Consistent time stamps and corresponding values in other columns.	✓	Same time resolution with little missing stamps
From_grid+production+discharge=consumption+charge	✓	Supply = demand
No double/duplicate records	✓	

### Validation level 2 & 3

Validation levels 2 is concerned with the check of consistency based on the comparison of the content of the file with the content of “other files” referring to the same statistical system (or domain) and the same data source.

- Consistency check with the “other files” can be other versions of exactly the same file. In this case the quality checks are meant to detect “revisions” compared to previously sent data.
- Consistency check with the “other files” can be versions of the same data set referring to other time periods. These checks are usually referred to as “time series checks” and are meant to verify the plausibility of the time series.

Below is the basic sanity check done in this work:

**Table:** 

**Expected**	**Received**	**Remark**
Work values file records = Measurement data file records	✗	Missing columns (from-grid, Feed-in) in measurement data file
Active power in measurement data = Energy in work values record	✓	Power and energy relation

### Data gaps

**Fig. 5:**
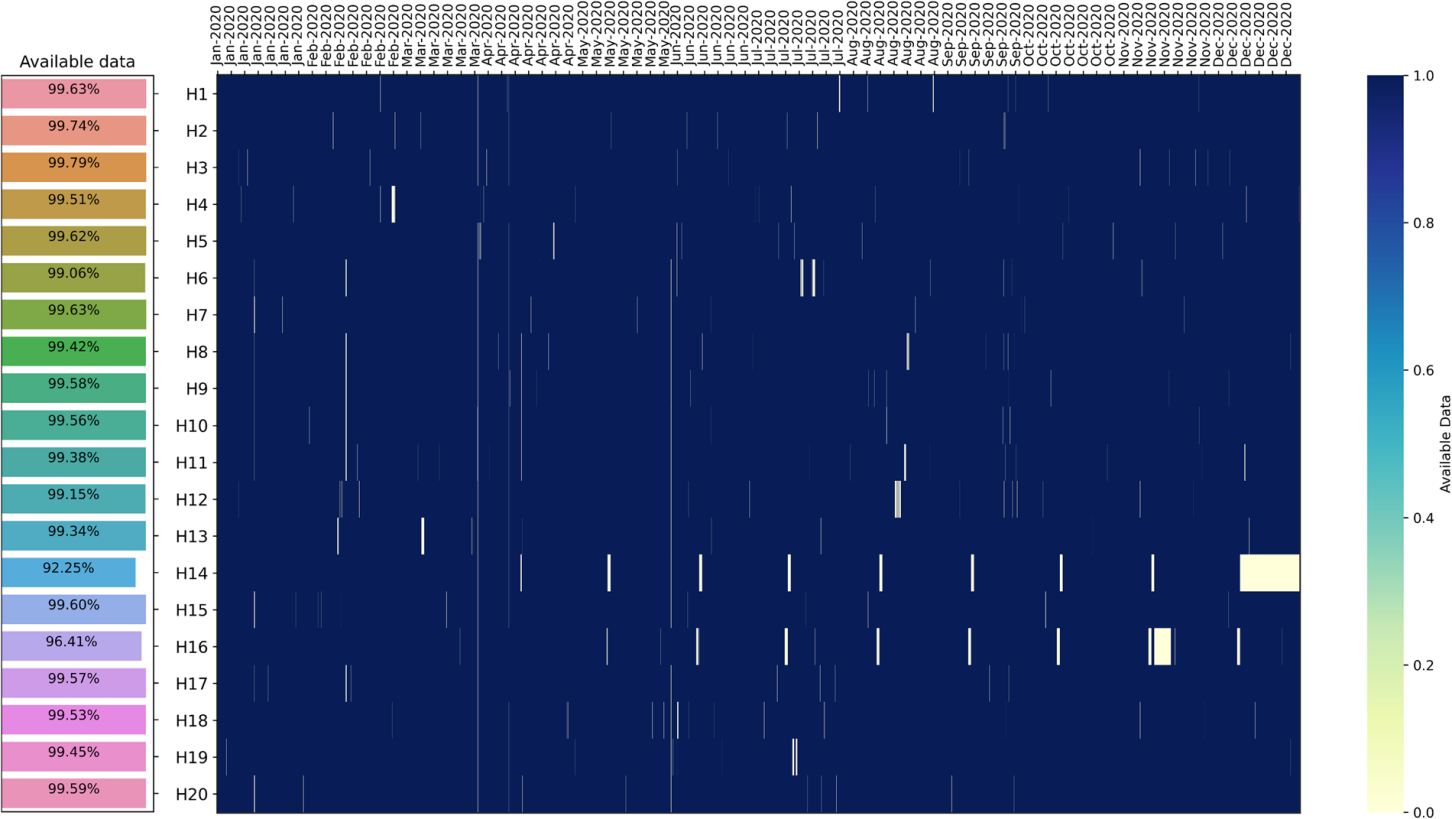
Data availability for all household measurements. Blue entries mark available data, white entries mark unavailable data. White zones appearing larger than one day are a visual phenomenon due to the limited number of pixels available for the plot and in reality, have data available in-between.

The chart in Fig. 5 shows the percentage of available data for 20 different houses in the year 2020. It also displays the times where data is missing on the timeline. Most of the data is available, with a data availability rate of over 99% for all houses except H14 and H16, which have availability rates of 92.25% and 96.41%, respectively.

Additionally, we discovered that within this 1-minute resolution datasets, and missing timestamps only occur for one or two consecutive minutes in a day. For example, for house H10, only two timestamps were missed on January 2nd, 2020, at 03:24 and 03:25. Throughout the entire year, 2317 timestamps were missed out of a total of 527039 timestamps, resulting in a 0.44% missing data rate. The main reasons for missing data could be technical data logging failures or internet disconnections leading to data loss. However, as the data gaps are small, we use linear interpolation between the nearest known values to fill in the missing data.

### Internal consistency of power and energy

The available datasets have both files for power and energy. For the graphical representation of validation on data consistency and quality, we randomly consider house H4, we validated the measurements with the available power and energy timestamps by constructing a plot wherein x-axis represents the measured power (W) for (a) production and (b) consumption and measured energy (Wh) for the same is represented on y-axis.

1 $$E=\underset{{t}_{1}}{\overset{{t}_{2}}{\int }}P(t)dt$$

**Fig. 6:**
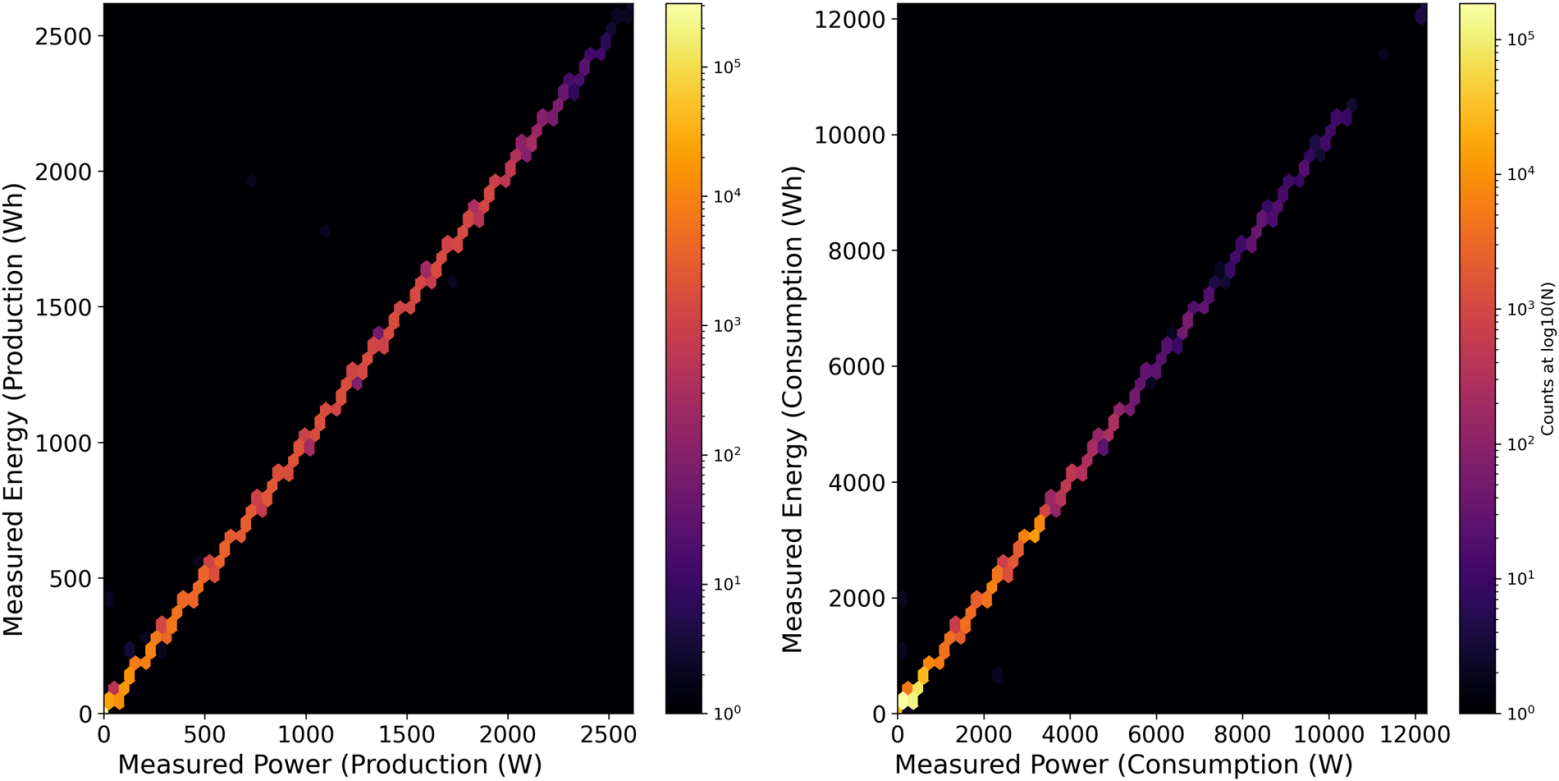
Comparison of measured Production & Consumption in power (W) and energy (Wh) for 1-minute interval households in the year 2020. We expect both measurement streams to be identical and mark this ideal state by a grey dotted diagonal. We plot both the colouring of the hexagons and the axes on logarithmic scales.

As shown in Eq. 1, E represents the total energy consumed, P represents the rate at which the energy is consumed between first time step t_1_ and last time step t_2_. This validation is performed on a resampled 1 min resolution dataset, as shown in Fig. 6. The colour of each hexagon is correlated with the number of timestamp points on the logarithmic scale. The Pearson coefficient of correlation for these two variables is 1.0, which means that there is an identical relationship between Wh and W. Overall, the measurements are highly correlated, and we did not identify any inconsistencies in the recorded measurements.

**Fig. 7:**
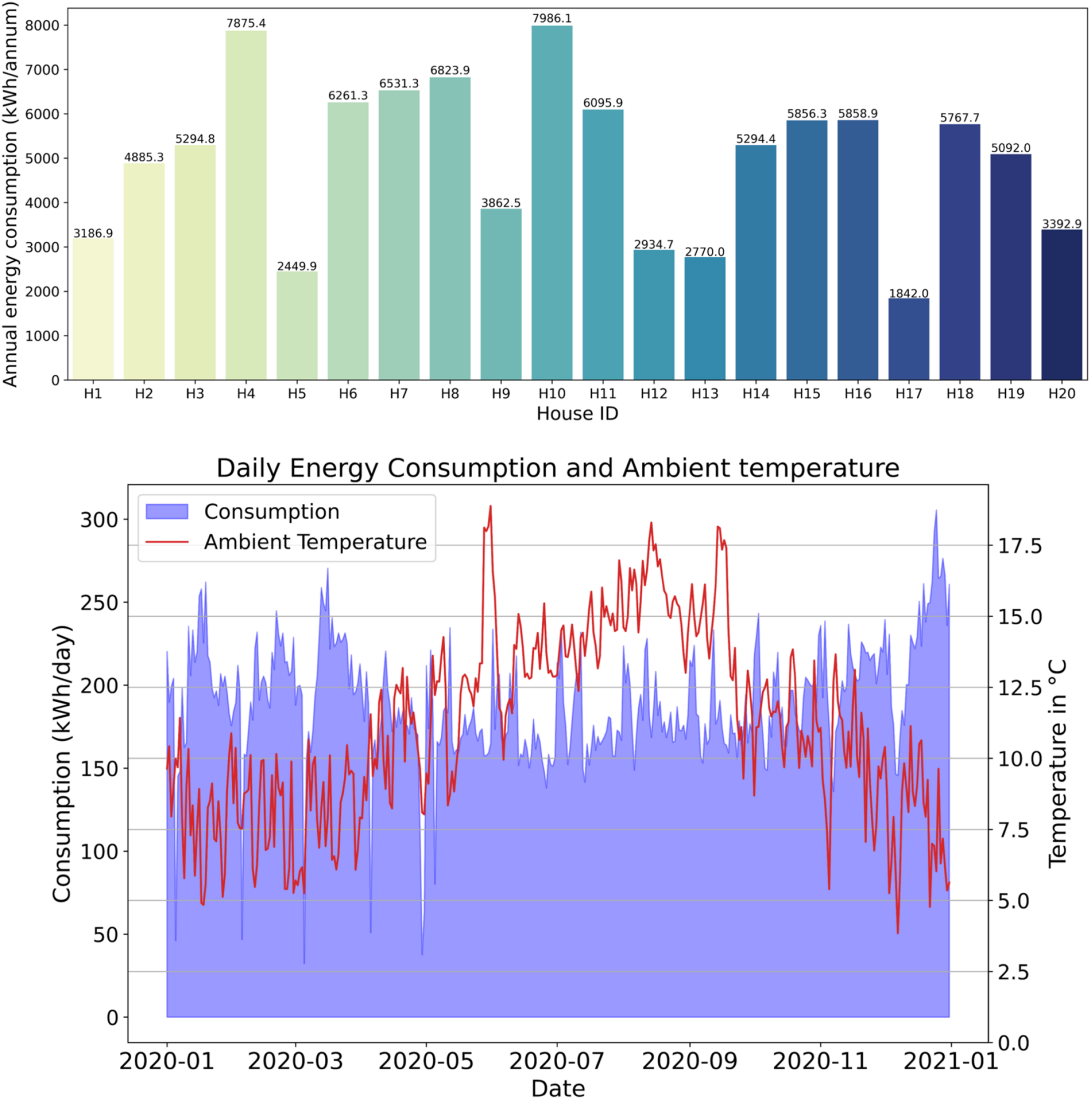
(**a**) Annual electricity consumption in 2020 for all households. The average electricity consumption of the households is 5025.64 kWh/annum, and (**b**) Daily Energy Consumption and Ambient Temperature.

Based on information from the Commission for Regulation of Utilities (CRU)^17^, the average annual electricity consumption for a 24-hour urban customer is 4,200 kWh. However, in our research conducted in a rural area on the West of Dingle Peninsula, the electricity consumption profiles varied due to factors such as the size of the house and the number of family members. Due to the GDPR issue, it was not possible to disclose the details of residents’ information/charecteristics. Their locations in the network have been identified and outlined in Fig. 2, including the sub-station transformer number. Thus, we could not learn more about the number of family members, their occupation, etc. We analysed the annual electricity consumption by 20 households (Fig. 7a), and collectively, their average annual electricity consumption came to 5025.64 kWh.

Figure 7(b) depicts the relationship between daily energy consumption (in kWh/day) and ambient temperature (in degrees Celsius, °C) for a group of 20 houses over a one-year period from January 2020 to January 2021. The temperature data is sourced from a weather station near the StoreNet project site in Dingle. The energy consumption for the aggregated 20 houses varies between approximately 0 and 300 kWh/day. Simultaneously, the ambient temperature fluctuates within a range of approximately 0 to 17.5 °C. Notably, there is an inverse relationship between energy usage and temperature: During warmer months, when temperatures are higher, energy consumption tends to decrease. Conversely, during colder months, when temperatures are lower, energy usage tends to increase. This correlation is supported by the Pearson correlation coefficient, which stands at −0.31. A negative coefficient indicates that as temperature rises, energy consumption declines, and vice versa. In summary, understanding these patterns can inform energy management strategies for residential areas, especially during extreme weather conditions.

**Fig. 8:**
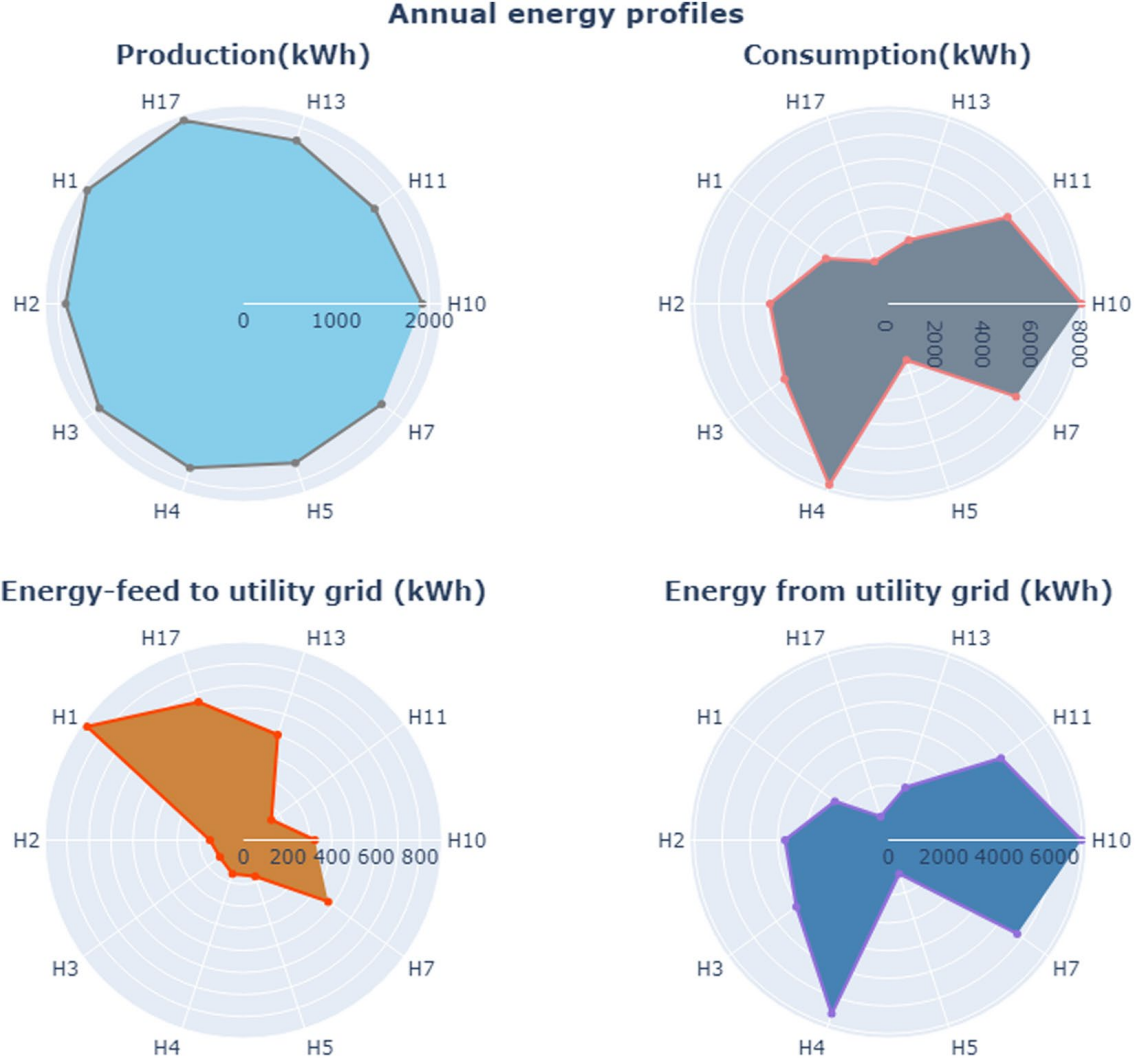
radar plot for annual energy profiles for houses with installed PV panels.

Figure 8 shows a radar plot summarizing the annual energy profiles of 10 out of 20 houses equipped with PV installations, along with their energy consumption and utility grid usage. According to the SEAI report^18^, a well-located solar PV system in a residential home with a capacity of approximately 3 kW can produce up to 2600 kWh of electricity annually. However, the actual electricity production is contingent on several variables, including the system’s size, hardware, geographical location, and the orientation of the panels. In the context of the StoreNet project, the installed PV capacity ranged between 2.0–2.2 kW. All these installations were oriented towards the south with an approximate inclination of 35 degrees. The Production radar plot clearly shows that all houses have PV generation within the 1800–2000 kWh range.

The energy exchange mechanism of these community houses can be validated in a straightforward manner. These houses are engaged in a strategy to maximize the consumption of their self-generated energy through the StoreNet project. For instance, house ID “H4” generates approximately 1900 kWh via its photovoltaic (PV) systems. However, its energy consumption demand is considerably high, around 8000 kWh. Consequently, it has to draw a substantial amount of power (approximately 6000 kWh) from the utility grid to meet its requirements, contributing only a minimal amount of energy back to the grid.

**Fig. 9:**
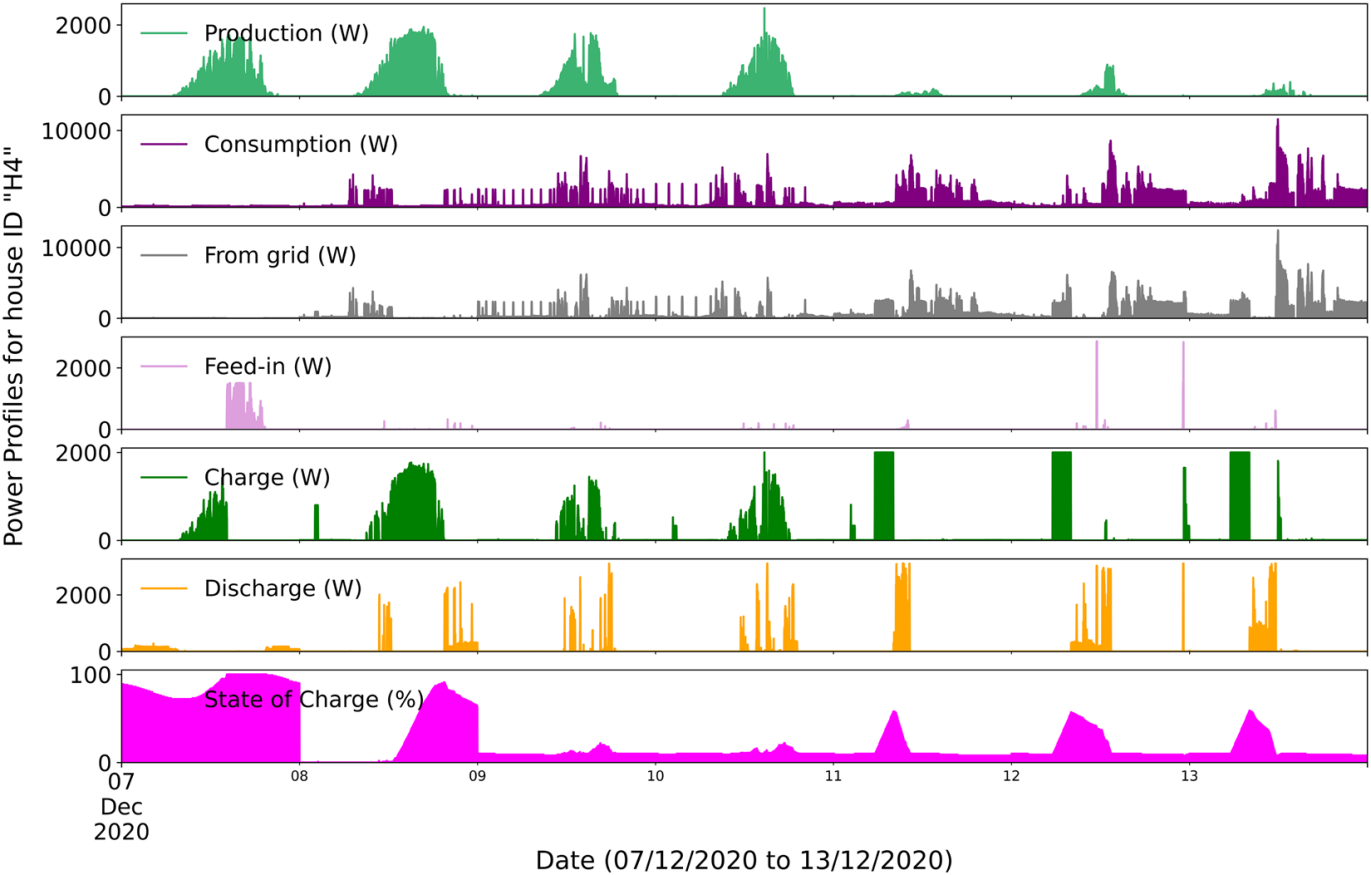
Daily pwoer profiles of production, consumption, grid geed-in, from grid and battery storage operation for house ID”H4”.

Figure 9 illustrates the power exchange profile for a household “H4” over a week ranging from 07th Dec to 13th December 2020. It shows a good overview of system balance information on high and low PV production days. Especially on 11th, 12th and 13th of December, the PV production is very low and hence there is more power imported from the grid. Similarly, on 8^th^ December, there was high PV production, but the consumption was very low. Hence, the battery uses part of the energy, and when it’s full, the grid feed-in occurs. This graph provides insights into energy dynamics within the household, balancing production, consumption, and storage.

Further validation of data was carried out in a research paper^19^ where real data from the StoreNet project in November 2020 was used to control battery storage operations. The study implemented a unique control approach in a system with batteries operating in a real-world environment. The aim of the proposed control solution was to minimize residential prosumers’ electricity bills, utilizing a hierarchical solution that combines an online and offline controller in the upper layer. Due to the challenges of tuning the local battery controller, a real-time controller was implemented in the cloud using the aggregator controller setup. The Irish day-night tariff scheme and a zero feed-in tariff were considered to evaluate the techno-economic merits of the solution. The field implementation results were verified through offline analysis by replicating the entire setup in an offline simulation environment (Table 3). The study found that the performance of the designed control system followed the operation in accordance with the data collected and presented in the research paper.

**Table 3:** Validation of data through simulation and measured by implementing bill minimisation control using a combination of offline controller (OffCtr) and real-time controller (RtCtr) proposed in the research paper^19^.

	Initial bill (Load expenditure) (€)	New bill (Consumption expenditure) (€)	Saving (%)
OffCtr + RtCtr (Simulation)	4.255	3.157	25.812
OffCtr + RtCtr (Measured)	4.255	3.168	25.557

Research on a peer-to-peer-based local electricity market for residential electricity end-users has been conducted using real-life measurements collected from StoreNet projects^20–22^. The research papers utilized the consumption profiles (without energy storage) of 20 households and solar PV generation profiles of 10 households for January 2020 (representative of winter) and June 2020 (representative of summer). Case studies for 55 residential households were conducted under these papers. The study utilized pre-processed data records of Sonnen energy consumption and production for these households in a standard IEEE EU LV distribution network. Hourly resolution profiles were obtained through averaging and missing data was filled using linear interpolation. Additionally, yearly measured data at half-hourly intervals were implemented in^23^ to investigate the possibilities of a real-life energy community (EC) in Ireland participating in a collective self-consumption (CSC) scheme. A blockchain-enabled platform has been developed to assess the technological, economic, and environmental impact of CSC managed by a community energy management platform for implementing real-life energy matching in EC.

## Potential use-cases of the dataset

**Fig. 10:**
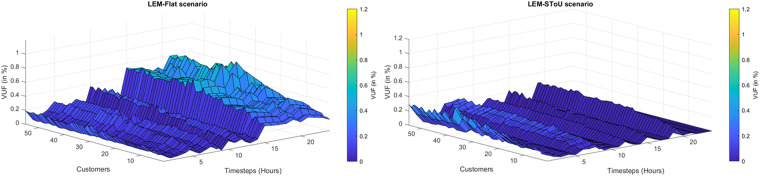
Voltage unbalance of all customer nodes for (**a**) LEM-Flat scenario on a winter day and (**b**) LEM-SToU scenario on a summer day.

- **Energy forecasting:** By analysing the data, researchers can develop models that predict energy consumption and generation patterns. These models can be used to optimise the grid’s operation and reduce energy costs.
- **Energy storage system analysis:** The dataset could be used to study the impact of energy storage systems on the grid. Researchers can develop strategies to optimise their operation and reduce energy costs by analysing the charging and discharging patterns of energy storage systems.
- **Microgrid or Community Self-Consumption analysis:** The dataset could be used to analyse the performance of microgrids or community self-consumption approach and develop strategies to optimise their operation. Researchers can study the impact of distributed energy resources (DERs) on the grid and develop strategies to ensure grid stability.
- **Renewable energy integration:** The dataset could be used to study the integration of renewable energy sources into the grid. By analysing the PV generation and grid import/export data, researchers can develop strategies to optimise renewable energy sources and reduce reliance on non-renewable sources.
- **Energy management:** The dataset could be used to study energy management in buildings. Researchers can develop strategies to optimise energy usage and reduce energy costs by analysing the active power consumption data.
- **Network impact: T**he dataset represents the energy profile of a community, including demand, PV generation, energy exchange with storage and grid. These can easily be implemented in any standard IEEE or CIGRE test bed networks^24^ to understand the impacts on grid networks due to the exchange of energy, such as peer-to-peer energy trading within the end-users in the community. As an example, Fig. 10 shows the impact of LEM due to retail market pricing on the voltage profile of the network^22^. Researchers can develop further control strategies depending on their needs and objectives to improve the network condition.

## Usage Notes

The StoreNet dataset is publicly available the data repository. The repository contains following folders and files, as given in Table 4:

**Table 4:** Data repository and its description.

Original data	‘Original data’ contains the data downloaded directly from the StoreNet project (File names are modified according to the battery number. It has 3 folders inside it: energy, power and weather. Energy folder contains all energy data (Wh) and power folder contains all power data (W). For e.g. filename “90956_2020_Wh” means, energy data for battery ID 90956 for year 2020. Weather data was added additionally downloaded from https://www.met.ie/climate/available-data/historical-data) and processed in one file for whole year.
processed_data	“processed_data’ contains the data which is mostly used for the data analysis and plotting figures. It has data which is resampled, interpolated, and arranged according to the need for plotting figures.
Figures	‘figures’ folder contains all the figures saved after running the python code.
data_processing.py	**data_processing.py** file is a python code that arranges data systematically, resample, interpolate for plotting purpose. Both **energy plots.py** and **power plot.py** files contain python code for generating figures. Some figures are generated using energy data and some using power data. Details and names of plots are in the commented section in these python files.
Energy plots.py	
Power plots.py	
Setup.py	‘setup’ folder contains all the dependencies to be installed before running the code. Alternatively, a user can install them separately using ‘pip install’ command for each dependency.

## Data Availability

A Python3.8 version was used to perform restructuring, validation and visualisation of the data, code of which is available at the data repository folder.
